# Efficacy and Safety of Combined Deferiprone and Deferasirox in Iron-Overloaded Patients: A Systematic Review

**DOI:** 10.7759/cureus.48276

**Published:** 2023-11-04

**Authors:** Ahmed Salem, Payal Desai, Ahmed Elgebaly

**Affiliations:** 1 Department of Pharmacology and Therapeutics, Levien Cancer Institute, Atrium Health, Charlotte, USA; 2 Department of Hematologic Malignancies and Blood Disorders, Levine Cancer Institute, Charlotte, USA; 3 Department of Internal Medicine, Wake Forest School of Medicine, Charlotte, USA; 4 Department of Medical Informatics, University of East London, London, GBR; 5 Department of Internal Medicine, Al-Azhar University, Cairo, EGY

**Keywords:** combination, deferiprone, deferasirox, iron overloaded, iron chelation therapy

## Abstract

Despite the established efficacy of iron chelation therapy in transfusion-induced iron-overloaded patients, there is no universal agreement regarding the choice of an optimal chelating regimen. Deferasirox (DFX) and deferiprone (DFP) are two oral iron chelators, and combination usage demonstrated effectiveness as an alternative to monotherapies in patients with a limited response to monotherapy. The present systematic review aimed to assess the evidence regarding the outcomes of combined DFP and DFX in iron-overloaded patients. An online search was conducted in PubMed, Scopus, Web of Science, and CENTRAL databases. Interventional and observational studies that assessed the outcomes of combined DFP and DFX in iron-overloaded patients were included. Eleven studies (12 reports) were considered in this meta-analysis. The studies included dual iron chelation strategies for a number of diagnoses. Single-arm studies (n =7) showed a reduction of serum ferritin, which reached the level of statistical significance in three studies. Likewise, most studies reported a numerical reduction in liver iron concentration (LIC) and increased cardiac MRI-T2* values after chelating therapy. Alternatively, comparative studies showed no significant difference in post-treatment serum ferritin between DFX plus DFP and DFX/DFP plus deferoxamine (DFO). The adherence to combination therapy was good to average in nearly 66.7-100% of the patients across four studies. One study reported a poor adherence rate. The combined regimen was generally tolerable, with no reported incidence of serious adverse events among the included studies. In conclusion, the DFP and DFX combination is a safe and feasible option for iron overload patients with a limited response to monotherapy.

## Introduction and background

Pathological accumulation of iron can cause serious complications, including cardiomyopathy, hepatic cirrhosis, endocrinopathy, and several other disorders, ultimately resulting in impaired organ function and potential organ failure [1]. Iron chelation therapy is a treatment modality for iron overload that eliminates iron from the body and effectively reduces the risk of related complications [2]. Three chelating medications are approved for treating iron overload and can be used as single agents or in combination. Parenteral deferoxamine (DFO) is an effective and relatively non-toxic chelating agent that normalizes heart function, particularly in the emergency setting [3]. However, DFO infusion occurs over 8-12 hours for 5-7 nights weekly, which makes it complex to administer, which can affect patients’ adherence and is expensive [2]. Further, the risk of DFO-related toxicities increases with overchelation [4]. Therefore, oral chelators emerged as effective and more convenient alternatives for DFO, including deferiprone (DFP) and deferasirox (DFX).

DFP is an oral chelator that is approved for iron overload in children and adults based on multiple clinical trials demonstrating its efficacy and safety. Literature suggests that the DFP can efficiently reduce serum ferritin; however, it is more effective when the baseline ferritin level is exceedingly high (>2500 ng/ml). Additionally, the impact of DFP on lowering liver iron concentration (LIC) is significant and comparable to that of DFO. Two randomized clinical trials [5,6] were conducted to compare the efficacy of DFP and DFO in lowering LIC; the results did not reveal any statistically significant difference between the two therapies. Furthermore, the superiority of DFP in removing myocardial iron has been reported in more than one study. Peng et al. [7] prospectively compared the effect of DFO and DFP on cardiac iron in 13 patients over a three-year period; cardiac iron was significantly improved in five DFP patients, compared to only two DFO patients. A larger, prospective controlled study [6] found that patients receiving DFP experienced significantly greater improvement in myocardial T2* than those receiving DFO, following a year of treatment (27% vs. 13%; p = 0.023). Likewise, multiple studies have shown that oral DFX reduces serum ferritin, LIC, and improves cardiac T2* in adults and pediatric iron overload patients. According to a randomized trial by Cappellini et al. [8], serum ferritin dramatically dropped over the course of a year as the DFX dose was gradually increased, with a mean reduction of 926 ng/ml, particularly in patients with high iron burdens. The ESCALTOR study yielded similar results, with a mean average reduction in LIC of 3.8 mg/g dry weight after one year of therapy with DFX [9]. In a three-year follow-up study, Pennell et al. [10] reported that myocardial T2* significantly increased from a mean of 12.0 ms to 17.1 ms, with 50% of patients experiencing improvements to 10-20 ms and 68% experiencing mT2* normalization.

The most common adverse events associated with DFP include gastrointestinal symptoms, zinc deficiency, and elevated transaminase levels. Agranulocytosis, although relatively infrequent with an incidence of 1%, represents a very serious side effect observed in individuals undergoing DFP treatment [11]. On the other hand, the most frequently reported DFX adverse events are gastrointestinal disorders, skin rash, high serum creatinine, and elevated liver transaminases. Other DFX-related adverse events like ocular and visual abnormalities, cytopenia, and Fanconi syndrome are less commonly reported [12]. Nonetheless, there is no universal agreement regarding the choice of chelating agents, and it largely depends on patient-related factors, local experience, physician preference, financial factors, and availability [13].

Despite the well-established efficacy of single-agent chelators, many patients demonstrate a limited response to a single drug and intolerance to increasing the dose to the maximum tolerated level. In such cases, combined therapy can represent a viable option for providing continuous coverage of chelating agents without increasing the risk of toxicity [14]. The DFO plus DFP combination is commonly used in clinical practice for patients with severe iron overload and has demonstrated efficacy in clinical trials [15]. However, as described above, such a regimen is complex and may limit patient compliance in non-emergency situations. Recently, combination therapy with DFX and DFP has been investigated and demonstrated efficacy in reducing iron overload and improving cardiac T2* without increasing the risk of adverse events [16]. Nonetheless, there is controversy over the efficacy of the DFX and DFP combination in iron overload patients, as the current reports show conflicting results regarding the efficacy of the DFX and DFP combination in severe iron overload [17-20].

The present systematic review aimed to assess the evidence regarding the outcomes of combined DFP and DFX in iron-overloaded patients.

## Review

Materials and methods

This systematic review was prepared in concordance with the PRISMA (Preferred Reporting Items for Systematic Reviews and Meta-Analyses) 2020 checklist [21].

Eligibility Criteria

Studies published between January 2010 and August 2022 were included if they fulfilled the following criteria: [1] studies that included pediatric and adult patients with iron overload who were resistant to chelating agent monotherapy; [2] studies that assessed the outcomes of combined DFP and DFX; and [3] studies that reported the change in serum ferritin level after treatment. There were no restrictions regarding the study design, sample size, or dosing regimen. We excluded preclinical and in-vitro studies, studies published in languages other than English, and conference proceedings.

Information Source, Search Strategy, and Selection Process

An online search was conducted in the PubMed, Scopus, Web of Science, and Cochrane Central Register of Controlled Trials (CENTRAL) databases from January 2010 to August 2022 using the following queries: ((("Iron Overload/drug therapy"[Majr])) AND "Deferiprone"[Mesh]) AND "Deferasirox"[Mesh]. Retrieved records were imported into EndNote X9 for duplicated removals. Two independent reviewers (AS and AE) then screened unique records in two steps for eligibility assessment: title and abstract screening, followed by selecting the full texts of eligible studies.

Data Collection Process and Risk of Bias Assessment

Two independent reviewers extracted prespecified data from eligible studies into a standardized Excel worksheet (Microsoft® Corp., Redmond, WA). The data included the year of publication, country, study design, population, sample size, reasons for selecting combination therapy, control group, dosing regimen and schedule, baseline characteristics of the patients (age, gender, and diagnosis), the change in serum ferritin after therapy, the change in LIC after therapy, the change in cardiac MRI-T2* values after treatment, and the rate of treatment-related adverse events. Two reviewers (AS and AE) assessed the risk of bias in included studies using the Cochrane risk-of-bias tool (RoB 2) for randomized trials [22] and the Newcastle-Ottawa Scale (NOS) for observational studies [23]. The overall judgment of the RoB 2 was either low, high, or some concern (i.e., unclear) about the risk of bias.

Results

**Figure 1:**
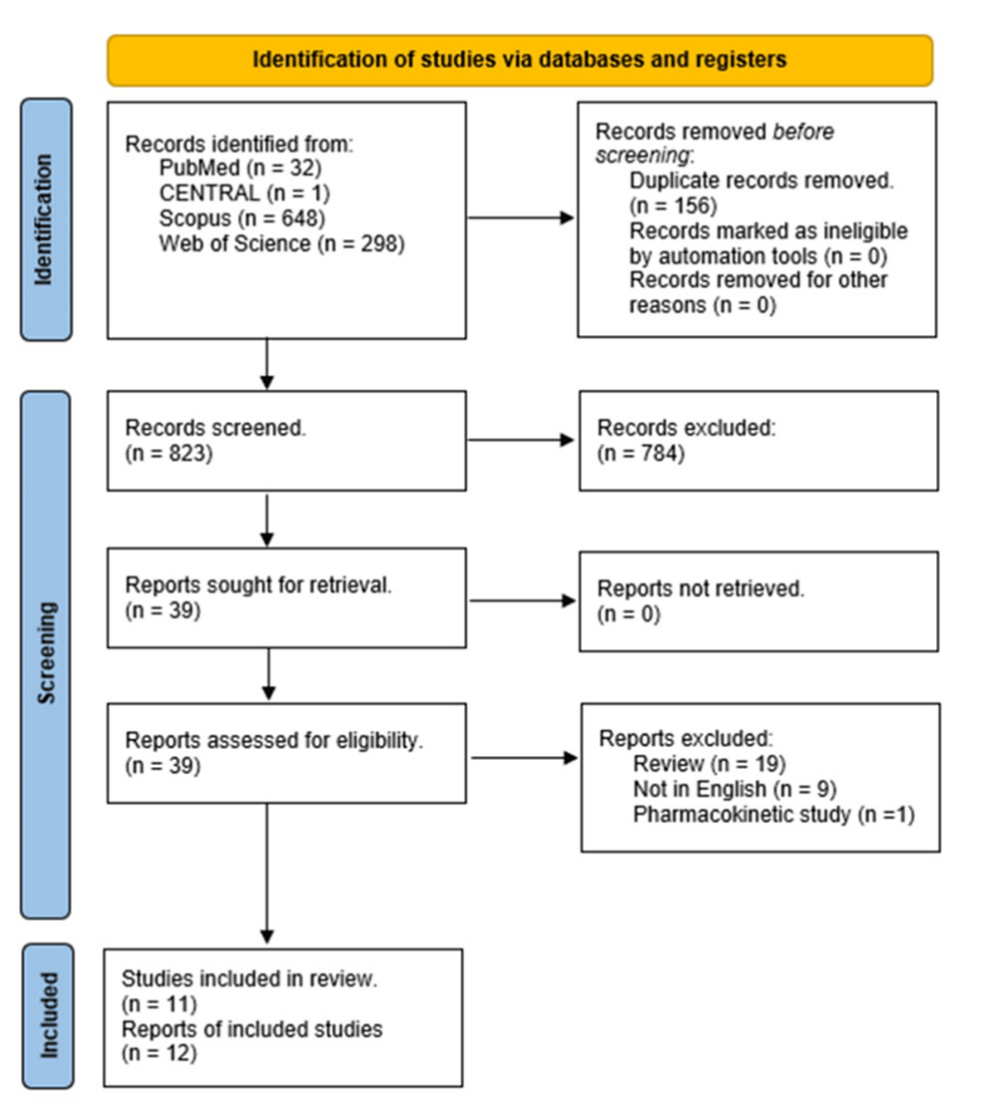
PRISMA 2020 flow diagram PRISMA: Preferred Reporting Items for Systematic Reviews and Meta-Analysis

A total of 823 unique records were retrieved and screened for eligibility. Of them, 39 articles were retrieved for full-text screening, and a total of eleven studies (12 reports) were deemed eligible (Figure 1).

Table 1 shows the summary characteristics of the included studies. Three included studies were open-label randomized controlled trials (RCTs) [16, 24, 25]. Three were prospective single-arm studies [26-28], one was a retrospective comparative study [29], and the remaining studies were case reports and series [17-20]. All studies included patients with transfusion-dependent thalassemia, and the sample size ranged from 1 to 96 patients. One comparative study compared DFX plus DFP and DFX plus DFO [29], one between DFX plus DFP and DFP plus DFO [16], one study compared the combined DFX and DFP with DFP monotherapy [25], and one study compared the oral combination with its mono components [24]. The main reasons for initiating DFX plus DFP across the included studies were suboptimal responses or intolerability to prior chelating therapy. The mean follow-up period of the included studies ranged from six to 36 months.

**Table 1:** Summary Characteristics of the Included Studies TDT, transfusion-dependent thalassemia; IOT, iron overload transfusion-induced; DFX, deferasirox; DFP, deferiprone; DFO, deferoxamine; mo, months; RCT, randomized controlled trial; SF, serum ferritin; NR, not reported; LVEF, left ventricular ejection fraction.

Author	Year	Country	Design	Population	Arms	Sample size	Reason for switching	Follow-up
Origa et al. [29]	2022	Italy	Retrospective Comparative	Adults and Children with TDT and Severe IOT	DFX plus DFP DFX plus DFO	58	Significant iron overload and refused DFO, or had local or systemic intolerance to it	36 mo
Jahir et al. [25]	2019	Bangladesh	Open-label RCT	Children with TDT	DFX plus DFP DFP	60	SF >1000 ng/ml	6 mo
Hammond et al. [28]	2019	USA	Prospective Cohort	Adults with TDT and Severe IOT	DFX plus DFP	9	SF >2500 ng/ml	18 mo
Pinto et al. [17]	2018	Italy	Case series	Untreatable TDT	Alternating DFX plus DFP	8	Intolerance	52 mo
Karami et al. [20]	2017	Iran	Case series	Adults with TDT	DFX plus DFP	6	NR	NR
Gomber et al. [24]	2016	India	Open-label RCT	Children with TDT	DFX plus DFP DFX DFP	49	SF >1500 ng/ml	12 mo
Elalfy et al. [16]	2015	Egypt and Oman	Open-label RCT	Children with TDT	DFX plus DFP DFP plus DFO	96	SF > 2500 μg/L	12 mo
Totadri et al. [27]	2015	India	Prospective Cohort	Adults and Children with TDT	DFX plus DFP	36	SF > 2000 μg/L	12 mo
Voskaridou et al. [18]	2011	Greece	Case report	Adults with TDT	DFX plus DFP	1	Significant iron overload	12 mo
Farmaki et al. [26]	2011	Greece	Prospective Cohort	Adults with TDT	DFX plus DFP	16	Intolerant to parenteral chelation with DFO	24 mo
Berdoukas et al. [19]	2010	USA	Case report	Adults with TDT	DFX plus DFP	4	Intolerant to parenteral chelation with DFO and excessive cardiac iron load with reduced LVEF	6 to 60 mo

Regarding the baseline characteristics of the included studies, the dosing schedule of the combined therapy was consistent across the included studies (DFP three times daily plus DFX once daily) in concordance with the approved label dosing, except in two studies. The combined therapy was administered in a daily alternating approach in the Pinto et al. study [17]. In the Elalfy et al. study, the DFP was administered twice daily at a fixed time (six- to eight-hour intervals) [16]. The pretreatment serum ferritin and LIC were notably high among the included studies (Table 2).

**Table 2:** Baseline characteristics of the included studies N, number of patients; LIC, liver iron concentration; DFX, deferasirox; DFP, deferiprone; TID, three times a day; QD, once a day; DFO, deferoxamine; SC, subcutaneous; NR, not reported; BID, twice a day.

Author	Year	Arm	N	Age	Male	Dose Frequency	Mean dose, mg/Kg	Duration of therapy	Serum ferritin, ng/mL	LIC, mg/g d.w.	cardiac T2* (m/sec)
Origa et al. [29]	2022	DFX+DFP	42	Adults 37±6; children 9±3	NR	DFP (TID)+DFX (QD)	DFP 93±13 DFX 23 ±5	95.8 patient-years	4031±2696	14.6±8.75	16±25
		DFX+DFO	16	38±6	5 (31.3%)	DFX (QD)+ DFO (SC)	DFX 23±7 DFO 42 ±8	44.1 patient-years	3472±1820	13.8±7.65	10.14±11.85
Jahir et al. [25]	2019	DFX+DFP	30	7.48±2.38	13 (43.3%)	DFP (TID)+DFX (QD)	DFP 68.68±4.84 DFX 27.98±2.1	6 mo	3413.70±1114.05	NR	NR
		DFP	30	7.42±2.50	22 (73.3%)	DFP (TID)	72.59±3.76	6 mo	3397.48±774.48	NR	NR
Hammond et al. [28]	2019	DFX+DFP	9	27.4	4 (44.4%)	DFP (TID)+DFX (QD)	NR		4965	28.5	13.3
Pinto et al. [17]	2018	DFX+DFP	8	28 (17–36)	4 (50%)	DFP (TID)+DFX (QD)	NR	52 mo	1632	NR	27
Karami et al. [20]	2017	DFX+DFP	6	23.8±5.8	5 (83.3%)	DFP (TID)+DFX (QD)	DFP 53.9±22.2 DFX 29.3±6.8	11.5±4.6 mo	2800±1900	7.59±3.16	16.69±15.35
Gomber et al. [24]	2016	DFX+DFP	15	11.6±6.21	30 (61%)	DFP (TID)+DFX (QD)	NR	NR	3696.5 (3079.6–4438.1)	NR	29.5±1.99
		DFX	17			DFX (QD)			3859.2 (3168.8–4700.0)	NR	32.0±2.00
		DFP	17			DFP (TID)			3140.5 (2617.5–3767.9)	NR	33.3±1.44
Elalfy et al. [16]	2015	DFX+DFP	48	14.05±2.21	32 (66.6%)	DFP (BID)+DFX (QD)	71.59±9.3 22.9±7.5	NR	4289.19±866.21	12.52±2.28	16.59±1.85
		DFX+DFO	48	15.25±2.31	30 (65.2%)	DFX (QD)+DFO (SC)	NR	NR	4379.07±895.00	12.69±2.23	16.32±1.82
Totadri et al. [27]	2015	DFX+DFP	36	13±6.9	26 (72.2%)	DFP TID+DFX (QD)	DFP+DFP 84.8±8.5 DFX 33.4±5.2	NR	6768±4145	NR	NR
Voskaridou et al. [18]	2011	DFX+DFP	1	34	0 (0%)	DFP TID+DFX (QD)	NR	12 mo	2080	NR	13.76
Farmaki et al. [26]	2011	DFX+DFP	16	35±7.5	9 (56.6%)	DFP TID+DFX (QD)	NR	NR	581±346	1.6±1.1	34.1±5.8
Berdoukas et al. [19]	2010	DFX (QD)+ DFP TID	3	17–22	NR	DFP TID+DFX (QD)	NR	NR	5170–11000	35–45	4.6±1.1

**Figure 2:**
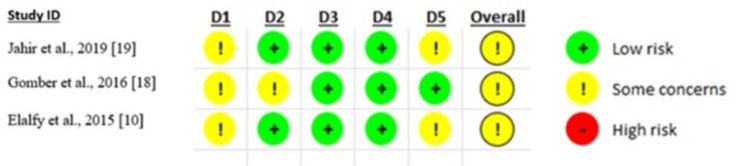
RoB assessment of the randomized controlled trials RoB: risk of bias

Three RCTs were assessed using the Cochrane RoB 2 and showed some concerns for the overall risk of bias assessment. The randomization processes were of some concern across the three studies, while one study had some concern regarding the risk of deviations from the intended interventions. All studies had low concerns regarding missing outcome data and measurement of the outcomes (Figure 2). The three observation studies had a moderate risk of bias, according to the NOS.

Efficacy of Combined DFP and DFX

Out of the 11 included studies, seven were single-arm reports evaluating DFP plus DFX in adults and children with transfusion-dependent thalassemia. Of them, three studies showed a significant reduction in the serum ferritin level 6-52 months after combined chelating therapy [18,26,27]. A total of 15 patients completed 24 months of treatment with both DFP and DFX; a notable reduction in the mean serum ferritin level, reaching statistical significance, was observed when compared to baseline (581±346 ng/ml vs. 103±60 ng/ml, p = 0.0001). Additionally, there was a significant decrease in LIC (p = 0.0019) and a significant increase in cardiac MRI T2* (p = 0.0381) among these patients [26]. Another study conducted on 32 patients who received the oral combination therapy for 12 demonstrated a statistically significant reduction in mean serum ferritin in 29 patients compared to baseline (3545±2907 ng/ml vs. 6768±4145 ng/ml, p = 0.0001) [27]. A 34-year-old patient who exhibited an inadequate response to the use of DFX monotherapy for three years, as evidenced by a baseline serum ferritin level of 2080 ng/mL and cardiac T2* of 13.76 ms, achieved a nearly normal serum ferritin level (397 ng/ml) and a normal level of cardiac T2* (21.1 ms) after receiving DFP and DFX treatment for one year [18]. In contrast, the remaining four studies showed a reduction in serum ferritin that did not reach the level of statistical significance [17,19,20,28].

Alternatively, two studies compared DFX plus DFP versus DFX/DFP plus DFO for a median follow-up of 12-36 months. In the study by Origa et al. [29], a total of 42 patients were treated with DFX plus DFP; the results showed a significant drop in serum ferritin levels after 12 months of treatment (4031± 2696 vs. 3259± 2235, p < 0.0001), and this reduction remained statistically significant at both the 24- and 36-month follow-up periods. Similarly, the LIC levels at the 36-month mark (9.73 ±6.61) demonstrated a significant reduction compared to the baseline assessment (14.6 ±8.75), particularly among those who maintained adherence to the prescribed therapy. These results demonstrated a resemblance to the results obtained from the comparable arm of the DFO plus DFX combination; specifically, there was no statistically significant difference between the two combination regimens in terms of the reduction of serum ferritin and LIC at the end of therapy. The same findings were reported by Elalfy et al., which compared the oral combination with DFO plus DFP [16]. After 12 months of therapy with the oral combination, serum ferritin levels were found to be significantly lower than baseline (3219.98 ± 882.25 vs. 4289.19 ± 866.21, p = 0.001), and mean LIC significantly decreased from 12.52 ± 2.28 to 10.17 ± 2.23, demonstrating results that align with the results observed in the DFO plus DFX comparison arm, with no significant difference observed in serum ferritin and LIC between the two cohorts. Additionally, a statistically significant increase in cardiac T2* was observed in the oral combination arm compared to the baseline (16.59±1.85 to 19.75 ms, P = 0.001), whereas the comparator arm did not demonstrate any significant change. One study compared the combined DFX and DFP with its mono components and showed that the combination therapy resulted in a significantly greater reduction in serum ferritin levels compared to DFP and DFX monotherapy (P = 0.035 and 0.040, respectively) after 12 months of treatment [24].

In terms of adherence, Origa et al. assessed the adherence according to the dispensed drug and found that 50% of the patients had good adherence (defined as the number of dispensed doses >80% of those prescribed) [29]. The favorable effect of the combination therapy was found to be dependent on adherence, as the reduction of serum ferritin was significant only when adherence to therapy was average-to-good, and, similarly, LIC decreased significantly in patients with good adherence (P = 0.006), whereas those with poor adherence did not exhibit any significant changes (p = 0.7). Berdoukas et al. reported that the good adherence rate was 66.7% (defined as excellent compliance with medication) [19]. Nevertheless, in the case of the patient who failed to completely adhere to the DFX component, an improvement in iron burden was noted. In the study by Pinto et al., the adherence rate was 90% (defined as the percentage of doses taken out of the total prescribed doses) [17]. The increased rate of adherence seen in this study may perhaps be attributed to the utilization of an alternating-agent regimen. On the contrary, Hammond et al. [24] reported a poor adherence rate of 56.7%. Despite the higher rate of non-adherence, there was a significant reduction in iron load among patients who were adherent to the assigned therapy. The study acknowledged that the non-adherence could potentially be driven by adverse events, particularly gastrointestinal symptoms, associated with the administration of the older DFX formulation (Table 3).

**Table 3:** Outcomes of the included studies *Donates statistical significance; LIC, liver iron concentration; MRI, magnetic resonance imaging; AE, adverse events; NR, not reported.

Author	Year	Time	Serum ferritin, ng/mL	LIC, mg/g d.w.	cardiac MRI-T2 (m/sec)	Adherence			Discontinuation		Adverse events				
						Good	Average	Poor	Improvement	AE or intolerance	Gastrointestinal discomfort	Elevated creatinine	Protein/creatinine ratio >0.5	Neutropenia	Vomiting
Origa et al. [29]	2022	Baseline	4031±2696	14.6±8.75	16±25	50.00%	33.33%	16.67%	11.90%	4.76%	14.29%	7.1%	9.52%	4.76%	0.00%
		Follow-up	3440±1860*	9.73±6.61*	14.73± 16.84										
Jahir et al. [25]	2019	Baseline	3413.70±1114.05	NR	NR	NR	NR	NR	NR	NR	0	NR	0	0	16.70%
		Follow-up	1654.20±934. 90*	NR	NR										
Hammond et al. [28]	2019	Baseline	5745±2517	27.8±14.1	15.5±9.6	56.7%			0	22.2%	22%	NR	NR	0%	33.3%
		Follow-up	4492±1077	30.2±15.4	14.9±7.3										
Pinto et al. [17]	2018	Baseline	1632	NR	27	90%			NR	NR	0	0%	0	0	0
		Follow-up	1045	NR	37										
Karami et al. [20]	2017	Baseline	2800±1900	7.59±3.16	16.69 ±15.35	NR	NR	NR	16.70%	33.30%	0	NR	0	16.67%	0
		Follow-up	3400±1600	5.41±2.22*	17.38±5.74										
Gomber et al. [24]	2016	Baseline	3696.5 (3079.6–4438.1)	NR	29.5	NR	NR	NR	NR	NR	NR	0%	0%	0%	0%
		Follow-up	2572.1 (2138.9–3093.1)*	NR	31.2±2.57										
Elalfy et al. [16]	2015	Baseline	4289.19±866.21	12.52±2.28	16.59±1.85	NR	NR	NR	NR	NR	12.50%	6.2%	0	10.40%	0
		Follow-up	3219.98±882.25*	10.17±2.23*	19.75±2.65*										
Totadri et al. [27]	2015	Baseline	6768±4145	NR	NR	NR	NR	NR	NR	11%	22%	25%	2.80%	0	0
		Follow-up	3545±2907*	NR	NR										
Voskaridou et al. [18]	2011	Baseline	2080	NR	13.76	100%	0	0	0	0	0	NR	0	0	0
		Follow-up	397*	NR	21.1*										
Farmaki et al. [26]	2011	Baseline	581±346	1.6±1.1	34.1±5.8	NR	NR	NR	NR	NR	20%	13%	0	0	0
		Follow-up	103±60*	1.0±0.2*	36.9±5.6*										
Berdoukas et al. [19]	2010	Baseline	5826	20.7	5.8±1.5	66.70%	0	33.30%	NR	NR	NR	NR	NR	NR	NR
		Follow-up	5544	28.1	7.0±1.5										

Safety of Combined DFP and DFX

The combined regimen was generally tolerable, with no reported incidence of serious adverse events among the included studies. In five studies, the incidence of gastrointestinal discomfort ranged from 12.2 to 22%. According to three of the five studies, combination therapy-induced gastrointestinal distress was typically moderate and transient and occurred at a rate comparable to that of monotherapy [16,26,27]. Four studies reported a serum creatinine increase in 6% to 25% of the participants; however, the levels remained within the normal range, therefore no corrective measures were taken [16,26,27,29]. Proteinuria (defined as a protein/creatinine ratio >0.5) was observed in two studies. In one study, it was temporary and resolved without interruption [26], whereas in the other, proteinuria was determined to be drug-induced only in three out of four reported cases [29]. In three studies, despite the low incidence of neutropenia (4.76-16.67%), it led to treatment interruption or dose reduction [16,20,29]. The occurrence of vomiting was documented in two reports, with a frequency ranging from 16.7% to 33.3% [25,28]. Elevated serum transaminases (ALT and/or AST) levels were reported in many of the included studies, with the majority of the reported incidents being mild, asymptomatic, and transient. However, Totadri et al. reported a substantial elevation in transaminases in four patients (11%), three of whom recovered after temporarily interrupting DFX, which was then resumed at a reduced dose, whereas in the fourth patient, DFX was not re-challenged because transaminases remained elevated [27]. Overall, the treatment discontinuation rate due to adverse events ranged from 4.8% to 33.3% across the included studies (Table 3).

Discussion

A single-agent iron chelator is generally an effective regimen for eliminating excess iron from the body in patients with iron overload. Nonetheless, a considerable proportion of the patients failed to achieve treatment targets with a monotherapy regimen or tolerate intensified doses of the single agents [30]. Therefore, combined regimens are commonly used in clinical practice for patients with severe iron overload who fail to respond to single agents. Recently, combination therapy with DFX and DFP has been investigated and demonstrated efficacy in reducing iron overload and improving cardiac T2* without increasing the risk of adverse events [16]. Combining DFX and DFP can lead to a synergistic effect of the two oral iron chelators and provide a convenient oral way of 24-hour coverage of iron chelation. In this systematic review, we found that a combined DFX and DFP regimen reduced serum ferritin, which reached the level of statistical significance in three studies. Besides, the LIC showed a numerical reduction, and cardiac T2* values showed a numerical increase. The combined DFX and DFP regimen was superior to its monocomponents and led to a similar efficacy profile to the combined DFO and DFP regimen.

Serum ferritin is widely used as a convenient and inexpensive serum marker for transfusion-induced iron overload [1,31]. In general, chelation therapy aims to achieve serum ferritin <1000 mcg/L. As previously mentioned, the combined DFX and DFP regimen led to an effective reduction in serum ferritin in most of the included studies in this systematic review. Moreover, comparative studies showed that the oral combination was more effective than the monocomponent and was as effective as DFX/DFP plus DFO, reflecting its feasibility as an effective and feasible alternative to conventional regimens. Although four studies in the present systematic review failed to reach the level of statistical significance in serum ferritin, this can be attributed to the small sample size and low precision. Notably, in Farmaki et al. [26], patients who were intolerant to DFO were included, despite the fact that their iron burden was not aggressive. The oral combination demonstrated safety and effectiveness in the included patients, opening the door for the initiation of combination therapy regardless of the burden of the disease. However, further cost-effectiveness investigations are required.

Non-invasive MRI measurement of LIC has become a common practice to detect liver iron deposition, assess its severity, and monitor treatment outcomes. LIC value estimated by MRI >7 mg/g dry weight indicates iron overload. A persistently high LIC (>15-20 mg/g dry weight) has been associated with adverse prognosis, liver fibrosis, and abnormal liver function [32,33]. Cardiac T2* has been the standard method for assessing myocardial iron deposition at the normal value of >20 milliseconds, with measurements below that value indicating iron overload. In contrast, values less than 10 milliseconds indicate significant myocardial iron deposition, which increases the risk of heart failure [34]. In the present systematic review, most studies demonstrated a trend toward a reduction in the LIC. Liver iron overload was reported to show a decreasing trend in the majority of the patients. Likewise, there was a trend towards an increase in the cardiac T2 value after combination therapy with DFP and DFX, highlighting that the DFP and DFX combination therapy could be used to alleviate cardiac and liver iron loading. Although the improvement in cardiac T2* can be attributed to the 24-hour coverage of the combination therapy, the DFX plus DFP combination may also exhibit a synergistic effect. Despite some studies showing that the improvement in the MRI values was not significant, the reduction in the three parameters was clinically relevant, and the insignificant results can be attributed to the small sample size of the studies.

It is now well established that continuous chelator coverage is essential to remove toxic labile iron that is constantly being generated [4,13]. Although the continuous infusion of the DFO can provide this 24-hour coverage, it is not a feasible option for most cases. Once-daily DFX has a plasma half-life of 12-18 hours and, hence, can provide sustained coverage of the labile iron within the body [35]. On the other hand, DFP is characterized by a low molecular weight that enables it to penetrate the cell for intracellular iron chelation [36]. Thus, a combined oral regimen of once-daily DFX and two/three-times daily DFP can lead to a convenient approach of 24-hour chelation coverage, higher efficacy in patients with severe iron overload, and better patient compliance. In particular, compliance is crucial for successful chelation therapy and has decreased the risk of iron-related morbidity and mortality [37]. Notably, in Elalfy et al. [16], the DFP was administered twice daily instead of the standard three times per day. Given the positive results of Elalfy et al., a twice-daily DFP may be able to provide 24-hour chelator exposure and can be considered to improve adherence in special situations. However, such an approach necessitates further evidence.

Four studies reported good-to-excellent adherence to the combined regimen in the present systematic review. In the study by Origa et al. [29] and Berdoukas et al. [30], 50% and 66.6% of the patients reported good adherence to the regimen. Pinto et al. [17] and Voskaridou et al. [27] reported overall adherence rates of 90% and 100%, respectively, reflecting the advantages of the combined DFX and DFP in improving adherence among patients with severe iron overload. The improvement in the adherence and satisfaction of the patients with the combination therapy can also be an indicator of the positive impact of the combined DFX and DFP on their quality of life. This was confirmed by Elalfy et al., who showed that the DFX+DFP combination was associated with a significant improvement in the quality of life of the patients [16]. According to Roberts et al., effective chelators significantly improve the quality of life and adherence of patients [38]. Ample evidence shows that DFO infusion was associated with impaired quality of life and that the quality of life of the patients improved after discontinuing DFO [26]. Alternatively, oral chelators were associated with improvements in all quality-of-life domains [39]. Therefore, it is expected that an effective combination of two oral chelators would lead to improvements in patient compliance and satisfaction.

Overall, the combination therapy was well tolerated across the included studies. Five studies reported gastrointestinal discomfort, with an incidence ranging from 12.5% to 22%. The incidence of neutropenia ranged from 0% to 16.67%, while vomiting was reported in only one study (16.7%). The incidence of elevated creatinine levels was low, and no agranulocytosis was reported, while only two serious adverse events led to discontinuation. While there is a theoretical expectation that the rise of transaminases would be additive due to the potential hepatotoxic effects of both drugs, most of the documented instances were of a mild nature, and the discontinuation of DFX alone was only necessary in one patient due to a persistent transaminase elevation. Such findings highlight the lack of additive side effects when using the combined regimen. Additionally, it was stated that the negative effects of this combination were the same as when the medications were administered alone, with no unanticipated additive side effects.

To the best of our knowledge, this is the first systematic review that has assessed the effectiveness and safety of the DFX plus DFP combination in iron-overloaded patients. Nonetheless, we acknowledge that the present study suffers from certain limitations. Most of the included studies had small sample sizes, which may affect the generalizability of our findings. Out of the 11 included studies, seven were single-arm and lacked control groups; thus, there was no control of potential confounders that might have affected the results of combination therapy. The overall quality of the included studies was generally poor, as indicated by the risk of bias in the assessment tools. All studies were conducted on transfusion-dependent thalassemia patients; hence, the generalizability of our findings to other iron-overloaded patients may be limited. Data regarding safety outcomes were inadequately reported in the included studies. Lastly, none of the included studies investigated the newer formulations of DFX and DFP, which is an area of future research.

## Conclusions

In conclusion, the DFP and DFX combination is a safe and feasible option for iron overload patients with a limited response to monotherapy. Patients with severe iron overload showed a significant reduction in serum ferritin and LIC and a significant increase in cardiac T2* values after the combined regimen. The combined regimen was associated with a high compliance rate and a well-tolerable safety profile. However, the current evidence is limited by the small sample size and low quality of the literature. Several issues regarding this combination are still unresolved, including the dosing schedule and efficacy of newer formulations of DFX and DFP, which require further research.
